# Notes and News

**Published:** 1893-11-25

**Authors:** 


### NOTES AND NEWS.

COLLECTED AND COMMUNICATED.

OLD students of St. Bartholomew's Hospital are reminded that the subscription list to the Andrew Testimonial Fund will be closed on the 1st December.

ERRONEOUS statements that the premises of Messrs. Cassell had suffered much in the late fire in Fleet Street have found circulation in the press. We are glad to be able to state that this is not the case. The danger was very imminent, and prompt arrangements for the printing of one periodical then in course of production were made. La Belle Sauvage has an excellent system of precaution against fire, and to this is due the smallness of the damage on Wednesday week. The Chief Superintendent of the London Fire Brigade paid the employés a high compliment for the assistance they rendered in preserving the premises from damage. It would be well on all large premises and in institutions if the inmates were instructed and practised in rendering assistance in case of fire.

WE are glad to see that attention is being called in the Press to the terrible danger of window-cleaning But whilst the methods at present employed prevail, and our windows are such as they are, it is difficult to see how the risk is to be minimized. The law has stepped in so far as the servant girl is concerned, but when other arrangements are made the use of a ladder is not always insisted upon. In THE HOSPITAL for June 3rd of this year we noticed the "Braloo window," an invention to obviate the inconveniences and dangers of window-cleaning. But this window is not altogether satisfactory, and scope yet remains for the inventor both to benefit himself and the whole of the community. We learn that nearly 100 deaths are caused in the United Kingdom in a year by standing and sitting outside windows to clean them. This is a large list of fatalities, the direct result of an occupation, and until some wise invention does away with the danger employers should be careful that no such calamities can be laid at their door, for with the employer undoubtedly much responsibility rests. In Vienna every employer has by police regulation to provide a safety belt for the use of window cleaners. This is attached by a cord to the casement. An amusing illustration of this belt in use is given in the *Daily Graphic* of the 21st inst. The illustration is hardly likely to add to the attractions of the useful belt.

THE Committee of the North-Eastern Hospital for Children, Hackney Road, have elected as their Secretary Mr. T. Glenton-Kerr, late Assistant Secretary at the Great Ormond Street Children's Hospital. Mr. Glenton-Kerr's record is good. He is highly spoken of by the authorities at Great Ormond Street, and we wish him all success in the difficult task he has so courageously undertaken.

A SPECIAL appeal for funds towards the endowment of a Pasteur Institute for India is now being instituted says the *Allahabad Pioneer*. It is proposed to locate this in some convenient place near Simla, where the necessary laboratories, fitted with the latest scientific appliances, will be provided, as also provisional accommodation for patients. The expenses for this erection will be very considerable, but the Government of India, besides giving their cordial approval to the scheme, have contributed notable help by promising the services of a selected medical officer. It is quite to be foreseen that now India has awakened to an interest in bacteriological work [illegible] this Institute may eventually extend itself in[illegible]ining school for practical demonstration in this [illegible]e, in addition to the anti-rabbic work to be carri[illegible] within its walls.

THE committee and secretary of the Surgical Aid Society are to be warmly congratulated upon a modification in the rules, which reflects the greatest credit upon their management, and proves their desire to really help the deserving. Formerly the letter system entailed much hardship upon the patients at times, as it was necessary to procure a large number of letters before surgical appliance could be obtained. We rejoice to see from the report of 1893, and from the new form of letter now used, that on satisfactory proof being given of the patient's inability to obtain all the letters, the secretary recommends the case for immediate benefit without further delay. During last year, out of 16,817 appliances supplied, 13,370 were given at once upon the production of the subscriber's letter. Even in respect to the more costly instruments and limbs the committee made 623 special grants under the new system. As this plan removes the chief objection to the former system of the Surgical Aid Society, we hope Mr. N. Tressidder, the secretary, will receive speedy and substantial proof of the giving public's appreciation and gratitude.

THE association formed to establish a national hospital for consumption in Ireland held their first general meeting recently. One of the facts most strongly impressed upon those present was that the scheme is no dead letter, but that progress towards the desired result is being steadily carried on. Already the working plans of the proposed building are completed, and the lease of the premises in County Wicklow only awaits Lord Fitzwilliam's signature. Lady Zetland, who from the first has been a most ardent supporter of the project, has consented to become patroness of the institution, which it is hoped shortly to commence. Invested funds amount to £8,500, there is a credit of £814 in the bank, and some £800 subscriptions promised have yet to be paid. We cannot doubt the success of so necessary an undertaking. Consumption hospitals are all too few in the British Isles, where the disease has so many victims. Each new institution has the advantage of the experience of its forerunners, and we can but hope that the committee of this important scheme will by obtaining the best expert opinions and employing competent hands to carry them out, embody in this Irish National Hospital all the virtues and none of the defects of existing institutions of its kind.

